# Severe Cytomegalovirus Congenital Infection With Neurological Compromise a Case Series Study in Mexico: Severe CMV and Neurological Compromise

**DOI:** 10.1155/2024/7510447

**Published:** 2024-10-17

**Authors:** Saúl Flores-Medina, Ricardo Figueroa Damian, Gabriela Arreola-Ramírez, Noemi Plazola-Camacho, Graciela Villeda-Gabriel, Sara A. Ochoa, Ariadnna Cruz-Córdova, Juan Xicohtencatl-Cortes, José Arellano-Galindo

**Affiliations:** ^1^Departamento de Infectología e Inmunología, Instituto Nacional de Perinatología, Ciudad de México, Mexico; ^2^CECyT 15 “DAE”, Instituto Politécnico Nacional, Ciudad de México, Mexico; ^3^Departamento de Seguimiento Pediátrico, Instituto Nacional de Perinatología, Ciudad de México, Mexico; ^4^Laboratorio de Investigación en Bacteriología Intestinal, Hospital Infantil de Mexico Federico Gómez, Ciudad de México, Mexico; ^5^Laboratorio de Inmunoquimica, Hospital Infantil de Mexico Federico Gómez, Ciudad de México, Mexico; ^6^Unidad de Investigación en Enfermedades Infecciosas, Hospital Infantil de México Federico Gómez, Ciudad de México, Mexico; ^7^Departamento de Medicina, Centro Interdisciplinario de Ciencias de la Salud Unidad Milpa Alta, Instituto Politécnico Nacional, Ciudad de México, Mexico

**Keywords:** cytomegalovirus, neurological compromise, PCR, symptomatic infection

## Abstract

Four cases of serious congenital cytomegalovirus (CMV) infections are described in this report. All cases were diagnosed postnatally using cerebrospinal fluid (3/4) or blood PCR (1/4) and histochemical study of the placenta (4/4). All infants were born prematurely. Maternal factors identified as significant were younger age at pregnancy and those from low-income social strata. The major clinical findings among patients with congenital CMV infection were hydrocephalus and persistent thrombocytopenia. The children's clinical condition did not improve over the course of the disease, leading to complications associated with extreme prematurity. Two of the children died, one of whom had severe brain malformations and showed neurological compromise at follow-up, seizures, motor impairment, and severe cognitive delay. It is essential to perform antenatal screening for possible CMV infection among pregnant women, even in countries with high population seropositivity, such as Mexico, to establish prenatal interventions to reduce the risk of fetal damage.

## 1. Introduction

Cytomegalovirus (CMV) is the most common cause of congenital infections around the world, both in developed and developing countries. In developing countries, such as Brazil, India, and Mexico, CMV seropositivity rates are higher than 90%, leading to congenital infection rates between 1% and 1.5%. In contrast, developed countries such as the United States and France have CMV seropositivity rates estimated between 40% and 50%, with the birth rate of congenital infection ranging between 0.3% and 0.5% [1, 2].

It is expected that most newborns infected with CMV and showing no signs or symptoms of CMV-associated disease will remain asymptomatic and not develop any long-term sequelae [3]. However, a small percentage of CMV-infected newborns will suffer from severe clinical manifestations and significant long-term adverse effects. Late diagnosis of congenital infection can significantly affect the quality of life of children and represent an emotional, economic, and social burden for the family and society as a whole. In this context, we present four cases of infants with congenital CMV infection who exhibited severe symptoms and developed serious sequelae, leading to functional impairment.

## 2. Cases Presentation

We are presenting a series of four cases, and the clinical characteristics of the mothers and infants are shown in Table 1. All of the infants were born prematurely; one of them was extremely premature, the other one was very premature, and two of them were moderately premature. The average delivery time was 31 weeks, the median was 30.3 weeks, and the maximum and minimum of gestational age (GA) were 36.4 and 21.1 of GA, respectively. In all cases, labor began spontaneously. The details of the cases are as follows.

### 2.1. Case 1

A female premature infant was born at 27.1 GA. She was admitted to the neonatal intensive care unit (NICU) and started on nasal CPAP for breathing support. However, she experienced apnea a few hours after admission and was intubated with assisted mechanical ventilation. During her neonatal period, she faced numerous health issues including hydrocephalus, thrombocytopenia, intraventricular hemorrhage, pulmonary hemorrhage, seizures, multicystic encephalopathy, patent ductus arteriosus, bronchopulmonary dysplasia, suspected sepsis, and retinopathy. On day 22, a transfontanellar puncture was performed to manage her hydrocephalus, and the cerebrospinal fluid (CSF) sample obtained indicated the presence of CMV DNA with a high viral load (VL). The serum levels of anti-CMV IgG and IgM were also positive. The pathological study of the placenta showed perivillous fibrinoid deposits plus dystrophic calcifications with a positive histochemical study for CMV. The patient was extubated on day 50 and left with nasal CPAP. She was discharged after 165 days of hospital stay. A follow-up was conducted when she was 5 years and 4 months old, which revealed that she had infantile cerebral palsy of the spastic diparesis type with limited mobility. She could walk but with limitations. Her manual skills were at level II, meaning she could handle most objects but with reduced quality and/or speed. According to Terman Merrill's intelligence evaluation, she had an overall score of 52 points, indicating mental retardation. She also experienced sporadic seizures. Her retina was considered normal, but she had strabismus.

### 2.2. Case 2

A female infant was born prematurely at 28.1 weeks of GA and showed signs of respiratory distress. She received pulmonary surfactant and was put on assist-controlled ventilation. Attempts to switch her to nasal CPAP were unsuccessful due to episodes of apnea, so she remained intubated for 63 days. Blood tests taken at birth showed low levels of white blood cells (2700 leukocytes/mm^3^) and platelets (59,000 platelets/mm^3^). On the fifth day of life, blood cultures were taken and came back positive for *Escherichia coli*, so she was treated with meropenem. However, her condition did not improve, and she was eventually diagnosed with septic shock, requiring the use of amines. The infant also had grade 2 bilateral intraventricular bleeding and status epilepticus, which were controlled with medication. On day 18 of life, a ventricular puncture was performed due to increased head circumference, and a viral panel study was conducted on the collected CSF, which tested positive for CMV (VL of 1 × 10^6^ genomes/mL). However, the serum anti-CMV IgM test was negative. The pathological study of the placenta showed chronic villitis and perivillous fibrinoid deposits, with a positive histochemical study for CMV. At 53 days of life, a ventricular access reservoir was placed due to increased hydrocephalus. The infant was discharged 131 days after birth and diagnosed with severe bronchopulmonary dysplasia, hydrocephalus, extensive cystic leukomalacia, and prematurity-associated retinopathy II. At 3 months of age, she showed poor responses to stimuli, motor autoregulation disorders, and frequent Moro reflexes but had adequate sucking, swallowing, and weak but consistent coordination. At 6 months of age, she was diagnosed with moderate-to-severe central coordination disturbances and a high risk of infantile cerebral palsy.

### 2.3. Case 3

A female infant was delivered via cesarean section. The prenatal ultrasound at week 31 indicated fetal neurological malformations and polyhydramnios. Further tests, including magnetic resonance imaging (MRI), confirmed that the infant had Dandy–Walker malformation with vermian dysgenesis and cerebellar hypoplasia, bilateral cleft lip, and occipital schizencephaly. Additionally, severe hydrocephalus and occipital subarachnoid cysts were also identified. Cesarean delivery was performed due to the fetus presenting breech. The infant was born late preterm at 36.4 weeks of GA with a birth weight of 3060 g, height of 48.5 cm, and head circumference of 44 cm. The infant experienced mild respiratory distress, which required oxygen therapy through cephalic helmets. At birth, the baby had several physical abnormalities, such as wide anterior and posterior fontanelles with a median cleft of the hard palate, micrognathia, low-set ears, right facial paralysis, and pectus excavatum. The baby had to undergo surgery to place a valve for a ventriculoperitoneal shunt. During this procedure, a sample of CSF was taken and sent to the laboratory for testing. The cytochemical report showed clear appearance, xanthochromic color, glucose 29 mg/dL, proteins 128 g/dL, negative for erythrocytes, one leukocyte per field, and few bacteria. The qPCR analysis for CMV in the CSF was CMV positive with a VL of 1 × 10^3^ genomes/mL. The serum anti-CMV IgG and IgM levels were also positive. The pathological study of the placenta showed chronic villitis, with a positive histochemical study for CMV. The baby also suffered from seizures. At 46 days, she presented with bronchospasm and a desaturation crisis and underwent nasopharyngeal ventilation, which did not produce an adequate response. She was then placed on mechanical ventilation with a laryngeal mask until her death, which occurred on day 61 of birth.

### 2.4. Case 4

A female infant born prematurely at 32.5 weeks was admitted to the NICU and needed ventilatory support since admission. The infant presented with jaundice, petechiae, and an enlarged liver of 4-4-3 cm below the costal margin, leukocytosis, thrombocytopenia, and eventually pulmonary hemorrhage. At 1 week of age, a positive serum anti-CMV IgM result was reported, and blood PCR was also positive. The placenta showed chronic villitis and perivillous fibrinoid deposits, with a positive histochemical study for CMV. Ganciclovir (6 mg/kg/day IV) was initiated at 15 days of age, and ophthalmological examination did not reveal chorioretinal lesions, but brain MRI showed ventriculomegaly, an increased diameter of the manga cistern, and leukomalacia. The infant remained intubated with high ventilatory parameters, and multiple organ failure was diagnosed. Unfortunately, the patient died on day 738 after birth. The results of the cytochemical studies performed on CSF specimens (taken at the time of PCR-confirmed CMV infection) are shown in Table 1.

In all cases, hypoglycorrhachia was observed, where evidence of an inflammatory process was inconclusive, as leukocyte values were within normal reference ranges. In only one case, a high level of protein was observed.

## 3. Discussion

Vertical transmission of CMV poses a significant challenge due to the difficulty in detecting maternal viral infection, as most infected pregnant women do not present any clinical signs. This results in unnoticed cases [4], as seen in the situations described in this paper, where none of the mothers underwent prenatal detection tests, leading to delayed diagnosis since their offspring already had severe complications. To prevent this, quarterly prenatal screening tests for CMV infection should be performed to enable early diagnosis during pregnancy, which could help identify fetuses at risk and establish appropriate intervention measures that reduce the risk of fetal developmental abnormalities [4, 5].

Low-income socioeconomic status and young age of pregnancy were the two most prominent maternal features in our patients, with 3/4 of mothers being less than 20 years old. These demographic data were found to be associated with CMV infection in a previous report, and in our patients, the same data were found in severe neurological infection [6].

In all cases, the premature birth was spontaneous. All newborns presented with hydrocephalus and thrombocytopenia, with their neurological function aggravated by intraventricular bleeding and seizures, which manifested as status epilepticus in 1 of the newborns as well as multisystem impairment and in three of them, these signs were attributable to their premature condition. However, the most serious signs have been reported, especially in premature newborns admitted to the NICU, who may present symptoms similar to sepsis and multisystemic alterations caused by CMV [7, 8]. One infant diagnosed CMV DNA positive in CSF was just at the same time clinically diagnosed with Dandy–Walker syndrome [9], suggesting thus plausible association between such syndrome and the viral infection. In our patients, the severity of the congenital infection was shown by the fact that 2 neonates died.

Children with severe congenital CMV infection may develop hearing impairment, motor deficits, speech delay, autism, attention deficit hyperactivity disorder, seizures, and vision problems, in addition to intellectual delay [10–12]. However, in the case of newborns without symptoms at birth, the prognosis is favorable with normal neurological development. Our follow-up showed that the two surviving children had severe motor and intellectual deficits, and approximately 60% of children with signs or symptoms at birth will develop long-term sequelae, mainly related to hearing and cognition problems [13]. Antiviral treatment for congenital infection is still a matter of debate, with no consensus on the usefulness of ganciclovir. In the United Kingdom, the use of this antiviral has been suggested on a case-by-case basis, and if used, it should be started before 4 weeks of life [14]. The prevention of vertical transmission is crucial, and although there is still little experience, valacyclovir can be used to diagnose acute CMV infection in pregnant women.

We have gathered data from several studies on the effects of CMV infection during pregnancy. One study showed that only 18% of newborns whose mothers had CMV and took valacyclovir during pregnancy had some symptoms of infection [15], compared to 57% of newborns whose mothers did not receive antiviral treatment. Another study found that pregnant women who received valacyclovir had a lower rate of CMV transmission to their babies than those who received a placebo [14–16].

In our study, we quantified the CSF VL in 3/4 patients with values between 2 × 10^3^ and 8 × 10^6^, and the use of CMV quantitation in CSF associated with CNS damage has been controversial, with a low prevalence of detection even in infants with CNS injury; however, there is a good correlation between CMV infection and CNS damage in cases where the virus was detected [17]. In our patients, with neurological compromise, we detect a considerable level of VL, showing that the CMV diagnosis by PCR in CSF can help to confirm the neurological infection in cases with severe neurological compromise.

The examination of the placenta can also be useful in diagnosing congenital CMV infection. In our research, we found that chronic villitis was the most common placental finding in the histopathological study, and histochemical study confirmed the presence of CMV infection. Other studies have reported similar findings, with chronic lymphoplasmacytic villitis and fibrotic, avascular villi being the most prevalent placental findings [18, 19].

## 4. Conclusions

This series of cases highlights the harmful effects of congenital CMV infection on the health of newborns who display symptoms. Infected children can suffer from multiple complications, some of which can even be fatal. Our findings stress the importance of implementing programs for pregnant women in prenatal care settings in countries like Mexico, where routine screening for CMV is not currently the norm. These programs can mitigate the impact of CMV infection and improve neonatal morbidity and mortality rates.

## Figures and Tables

**Table 1:** Sociodemographic and clinical characteristics of mothers and their children.

**Case**	**1**	**2**	**3**	**4**
Mother				
Age (years)	28	15	18	19
Occupation	Retailer	Housewife	Housewife	Housewife
Gestation number	2	1	2	1
Clinical status	Asymptomatic	Asymptomatic	Polyhydramnios	Asymptomatic
Pregnancy comorbidities	Cystitis	Drug addiction	Obesity	None
Pregnancy outcome	Preterm labor	Preterm labor	Preterm labor	Preterm labor
Child				
Age of gestation	27	28.1	36	32
Sex	Female	Female	Female	Female
Birth weight (g)	900	960	3060	1680
Head of circumference	28	26	44	30
Symptomatology associated to CMV	Prematurity hydrocephalus thrombocytopenia	Prematurity hydrocephalus thrombocytopenia	Multiple brain prematurity and cerebellum malformations, hydrocephalus, late	Prematurity, ventriculomegaly jaundice, petechiae, hepatomegaly. Premature thrombocytopenia
Clinical condition at birth	Neonatal asphyxia requiring intubation and mechanical ventilation	Neonatal asphyxia requiring intubation and mechanical ventilation, seizure crisis	Required ventilatory support CPAP	Without respiratory effort leading to intubation. Apgar 3/5
Final outcome	Severe psychomotor delay. Seizure crisis	Severe psychomotor delay. Seizure crisis	Mechanical ventilation and death on day 60	End-organ disease and death on day 38
Viral load in CSF (genomes/mL)	1 × 10^6^	1 × 10^6^	1 × 10^3^	Nondeterminated

## Data Availability

The data that support the findings of this study are available on request from the corresponding author.
